# Long DCL4-substrate dsRNAs efficiently induce RNA interference in plant cells

**DOI:** 10.1038/s41598-019-43443-9

**Published:** 2019-05-06

**Authors:** Sayaka Kakiyama, Midori Tabara, Yuki Nishibori, Hiromitsu Moriyama, Toshiyuki Fukuhara

**Affiliations:** 1 Department of Applied Biological Sciences, Tokyo University of Agriculture and Technology, 3-5-8 Saiwaicho, Fuchu, Tokyo 183-8509 Japan; 2 Institute of Global Innovation Research, Tokyo University of Agriculture and Technology, 3-5-8 Saiwaicho, Fuchu, Tokyo 183-8509 Japan

**Keywords:** Molecular engineering in plants, Molecular engineering in plants, Plant molecular biology

## Abstract

RNA interference (RNAi) is induced by the direct transfer of double-stranded RNAs (dsRNAs) into protoplasts prepared from *Arabidopsis thaliana* seedlings. In this protoplast RNAi system, we compared the efficacies of various-sized dsRNAs (between 21 and 139 nucleotides [nt]) for inducing RNAi and assessed the dsRNA-cleaving activities of Dicer-like 3 (DCL3) and 4 (DCL4). After the direct transfer of dsRNAs into protoplasts, cleaved RNA products of 21 nt were detected from long 130- or 500-nt dsRNAs by DCL4 but not from 37-nt dsRNAs. These results indicate that DCL4 preferentially cleaves long dsRNAs in protoplasts, consistent with our previous biochemical data regarding the substrate specificity of DCL4. Direct transfer of long dsRNAs of approximately 130 nt into protoplasts induces RNAi much more effectively (by approximately 60- to 400-fold) than direct transfer of short 37-nt dsRNAs. Although transfer of 21-nt dsRNAs into protoplasts induced RNAi without DCL4 activity, the induction of RNAi was less effective (by approximately 0.01-fold) compared with long dsRNAs. These results indicate that cleavage of long dsRNAs exceeding 100 nt by DCL4 into 21-nt dsRNAs is essential for efficient induction of RNAi in plant cells.

## Introduction

RNA interference (RNAi) is the process of sequence-specific post-transcriptional gene silencing (PTGS) triggered by double-stranded RNAs (dsRNAs)^1,2^. In the RNAi pathway, a Dicer endoribonuclease cleaves long dsRNAs into small interfering RNAs (siRNAs)^3^, which then associate with the protein Argonaute (AGO)^4^. siRNA-loaded AGO binds specific mRNAs with sequence-complementarity, cleaving them or inhibiting their translation^5^.

Most eukaryotic organisms except for mammals, including fungi, insects, and plants, which have no antibody-mediated immune system, sense long exogenous dsRNAs as viruses and then activate the RNAi (PTGS) pathway for defence against virus infection^6–8^. In contrast, mammals sense long exogenous dsRNAs as virus infections and respond by activating the immune system, which has specifically developed innate and adaptive immune components for defence against pathogens^9,10^. Therefore, dsRNAs longer than 30 nucleotides (nt) are generally used to induce RNAi in fungi, insects and plants^1,2,11^, but in mammals, they often activate the innate immune system including the interferon pathway^9,10^. Shorter dsRNAs (siRNA duplexes) of 20 to 22 nt in length are typically used in RNAi experiments, as they prevent activation of the immune response in mammalian cells^12^. Furthermore, Kim *et al*. demonstrated that dsRNAs of 25 to 30 nt in length induce RNAi as much as 100-fold more potently than 21-nt dsRNAs (siRNA duplexes), and this greater potency depends on processing of the 25- to 30-nt dsRNAs by Dicer^13^. They termed these dsRNAs “Dicer-substrate” siRNAs (dsiRNAs).

Although direct transfer of dsiRNAs or siRNAs into cultured cells is a standard protocol in RNAi experiments in mammals^12,13^, the direct transfer of dsRNAs into intact plant cells is difficult because of the cell wall. Therefore, because transgenic plants have been easily created since the 1980’s, researchers have studied RNAi in plants by creating transgenic plants that express stem-loop (fold-back) single-stranded RNAs (ssRNAs) containing a long dsRNA region of 100 to 1,000 nt^14^.

In plants, many important genes involved in RNAi have been discovered and characterized in genetic studies using mutants of the transgenic RNAi-expressing model plant, *Arabidopsis thaliana*^15,16^. However, characterizing the enzymatic properties of proteins that constitute the RNAi machinery in plant cells is difficult, because plant cells usually have a large central vacuole containing a variety of proteinases and nucleases. The biochemical properties of two core proteins (Dicer and Argonaute) in the RNAi machinery have been extensively studied in humans, the fruit fly *Drosophila melanogaster*, and the nematode *Caenorhabditis elegans*^17–19^. Therefore, even though some important genes encoding RNAi machinery components were first discovered in the model plant *A*. *thaliana* via genetic studies^15,16^, findings from biochemical studies related to the molecular mechanism of plant RNAi are limited.

We established a simple biochemical method to simultaneously monitor the dsRNA-cleaving activities of Dicer-like 3 (DCL3) and 4 (DCL4) in cell-free extracts of *Arabidopsis* seedlings^20–23^. We demonstrated that the enzymatic properties of DCL3 and DCL4 differ markedly *in vitro* and that they preferentially cleave short (<50 nt) and long (>50 nt) dsRNAs, respectively^20,21^. The dicing activities of DCL3 and DCL4 are post-translationally regulated by inorganic phosphate and the redox state^22^. Our results from *in vitro* biochemical studies are consistent with reports indicating that RNA polymerase IV (Pol IV)-dependent RNAs, which are the precursors of 24-nt siRNAs produced by DCL3, are short ssRNAs of 30 to 40 nt in length *in vivo*^24,25^. Our previous biochemical results are also consistent with the *in vivo* function of DCL4, in which the enzyme primarily cleaves long viral dsRNAs into 21-nt viral-derived siRNAs (vsiRNAs) in most virus-infected plant cells^26,27^.

It is difficult to evaluate the efficacies of various-sized dsRNAs in terms of inducing RNAi using transgenic plants expressing stem-loop ssRNAs containing a long dsRNA region^14^. Consequently, in this study, we evaluated the efficacies of various dsRNAs for inducing RNAi by direct transfer of dsRNAs into protoplasts prepared from *Arabidopsis* seedlings. Using this protoplast RNAi system, we compared the efficacies of various-sized dsRNAs for inducing RNAi and assessed the dsRNA-cleaving activities of DCL3 and DCL4 for long and short dsRNAs as substrates in *Arabidopsis* protoplasts. We demonstrated that DCL4 preferentially cleaves long dsRNAs (>100 nt) in protoplasts. Moreover, these long DCL4-substrate dsRNAs induce RNAi as much as 400-fold more potently than 21- or 37-nt dsRNAs. Therefore, the cleavage of long dsRNAs by DCL4 is essential for the efficient induction of RNAi in plant cells.

## Results

### DCL4 preferentially cleaves long dsRNAs into 21-nt RNAs in protoplasts

**Figure 1:**
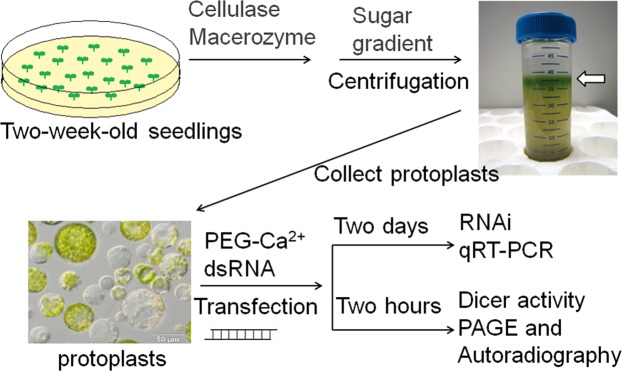
Schematic representation of the experimental procedure to examine the efficacies of various dsRNAs for inducing RNAi. Various dsRNAs were directly transferred into protoplasts prepared from 2-week-old *Arabidopsis* seedlings, and then their efficacies for inducing RNAi were analysed using qRT-PCR. DCL3 and DCL4 activities were analysed by denaturing PAGE and autoradiography. For details see Methods^28,29^.

**Figure 2:**
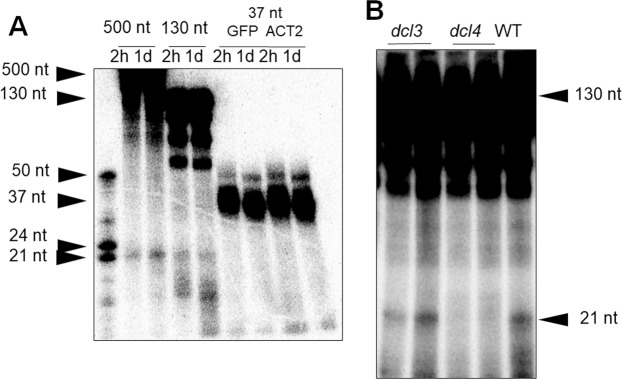
The dsRNA-cleaving activity of DCL4 in *Arabidopsis* protoplasts. Protoplasts prepared from 2-week-old *Arabidopsis* seedlings were transfected with ^32^P-labeled dsRNAs of 37 nt (**A**), 130 nt (**A**,**B**), or 500 nt (**A**). Total RNAs were extracted from protoplasts 2 hours (**A**) or 1 day (**A**,**B**) after transfection, and electrophoresed by denaturing 15% PAGE. Cleaved products were detected by autoradiography. Protoplasts were isolated from WT (**A**,**B**), *dcl3* (**B**), or *dcl4* (**B**) seedlings, and the 37-nt dsRNAs (**A**) were synthesized from the *ACT2* and *GFP* genes and dsRNAs of 130 nt and 500 nt were synthesized from the *GFP* gene. Two biological replicates are shown for lanes *dcl3* and *dcl4* in (**B**).

We established a simple biochemical method to simultaneously monitor the dsRNA-cleaving activities of DCL3 and DCL4 in cell-free extracts of *Arabidopsis* seedlings and demonstrated their enzymatic properties *in vitro*^20–23^. To monitor and characterize the dsRNA-cleaving activities of these Dicer enzymes in plant cells, we prepared protoplasts from 2-week-old seedlings of *A*. *thaliana*, transfected protoplasts with various ^32^P-labeled dsRNAs as substrates, extracted total RNAs, and then analysed cleaved RNA products using denaturing polyacrylamide gel electrophoresis (PAGE) and autoradiography (Figs 1 and 2). In protoplasts transfected with 130- and 500-nt dsRNAs, ^32^P-labeled 21-nt RNAs were detected, but no small RNA products ranging from 21 to 24 nt were detected in protoplasts transfected with 37-nt dsRNAs (Fig. 2A). Based on size, the ^32^P-labeled 21-nt RNAs were expected to be DCL4 cleavage products. As no cleaved products were detected in protoplasts prepared from *dcl4* mutant seedlings (Fig. 2B), they were confirmed as products of DCL4 cleavage. These results indicate that DCL4 preferentially cleaves long dsRNAs (>100 nt) into 21-nt RNAs in protoplasts (plant cells), confirming our previous finding demonstrating that DCL4 preferentially cleaves long dsRNAs into 21-nt dsRNAs *in vitro*^21^. However, we could not detect activity of DCL3 (which produces 24-nt siRNAs) in protoplasts (Fig. 2) even though the dsRNA-cleavage of short 37-nt substrate dsRNAs by DCL3 was easily detected in an *in vitro* biochemical assay^21^. Although the weak dicing activity of DCL4 to 37-nt dsRNAs was also detected in an *in vitro* assay^21^, we could not detect 21-nt RNA products from 37-nt dsRNAs by DCL4 (Fig. 2A). The results indicated that the sensitivity to detect the dicing activity in protoplasts is lower than that in an *in vitro* biochemical assay^21^.

### Long dsRNAs of approximately 130 nt induce RNAi much more effectively than short dsRNAs of 37 nt

**Figure 3:**
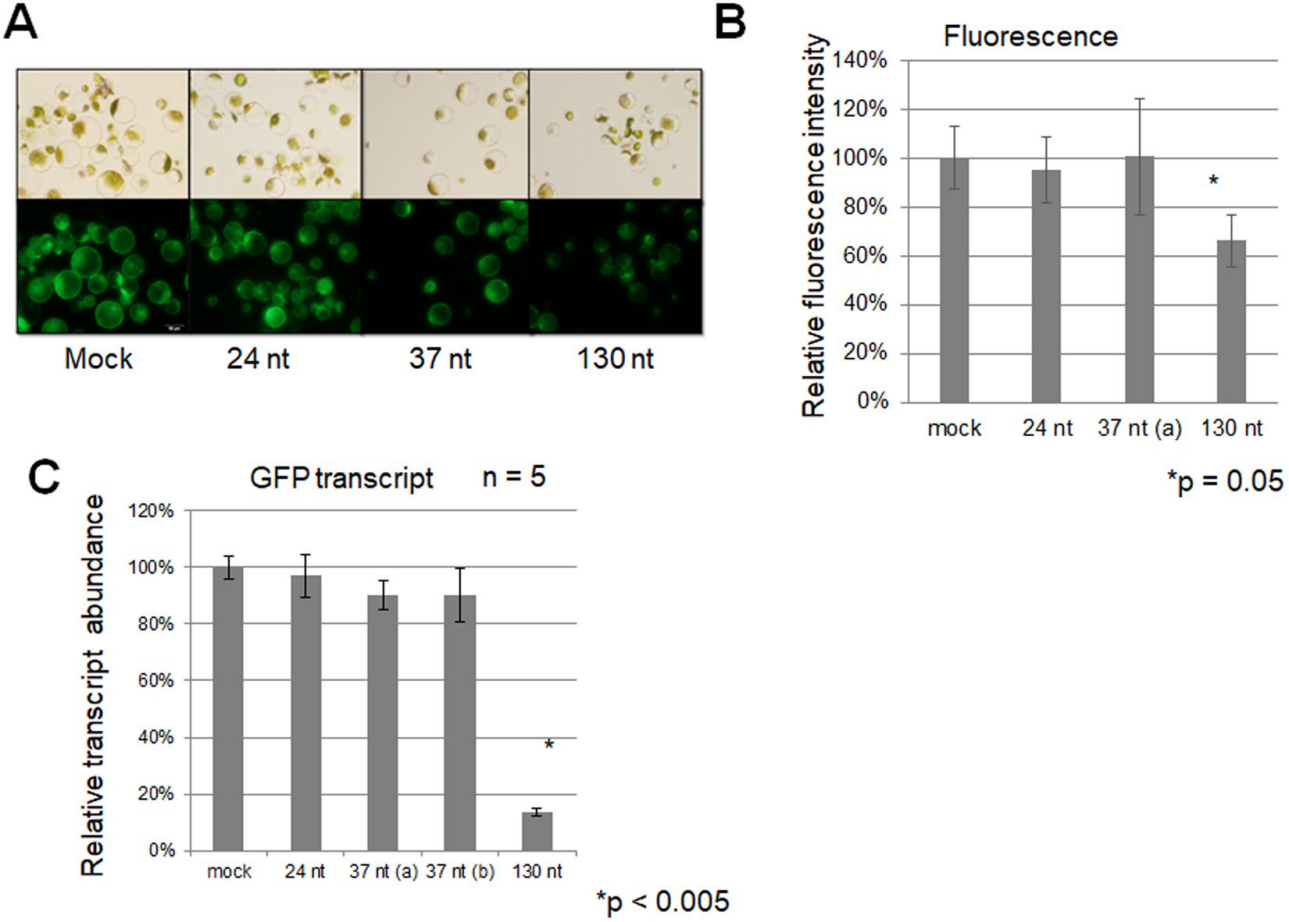
RNAi (gene silencing) for the exogenous GFP gene was successfully induced by direct transfer of long dsRNAs of about 130 nt into protoplasts. Approximately 3 µg of dsRNA was transfected into 0.1 mL of protoplasts (10^6^ to 10^7^ cells/mL) prepared from 2-week-old *Arabidopsis* seedlings over-expressing the *GFP* gene. The efficacies of various-sized dsRNAs for inducing RNAi were evaluated by (**A**,**B**) observing green fluorescence by microscopy and (**C**) analysing the relative abundance of *GFP* transcripts by qRT-PCR. (**A**) Bright-field (upper panels) and fluorescence (lower panels) micrographs of protoplasts transfected without (mock) or with 3 µg of various-sized dsRNAs against GFP (RNAi) were examined, and the fluorescence was quantified. Fluorescence micrographs were photographed 2 days after transfection. All images were captured for the same exposure period. (**B**) Fluorescence intensities of dsRNA-transfected protoplasts relative to that of mock-transfected protoplasts (100%) were calculated using ImageJ software. Error bars indicate the standard errors of three replicates, and the p-value was determined using the Student’s *t*-test. (**C**) The relative abundance of *GFP* transcripts in dsRNA-transfected protoplasts compared to mock-transfected protoplasts (100%) was determined by qRT-PCR. Protoplasts in 100 µL (10^6^ to 10^7^ cells/mL) were transfected without (mock) or with 3 µg of various-sized dsRNAs against the exogenous GFP gene (RNAi). Two 37-nt dsRNAs (a and b) with a different nucleotide at each end were used. Total RNAs were extracted from protoplasts 2 days after transfection. The efficacies of dsRNAs for inducing RNAi were analysed by qRT-PCR for the relative abundance of *GFP* transcripts normalized to *ACT2* transcripts (house-keeping gene). Error bars indicate the standard errors of five biological replicates, and p-values were determined using the Student’s *t*-test. Asterisks (*) in (**B**,**C**) indicate significant differences between mock and 130-nt dsRNA transfections.

**Figure 4:**
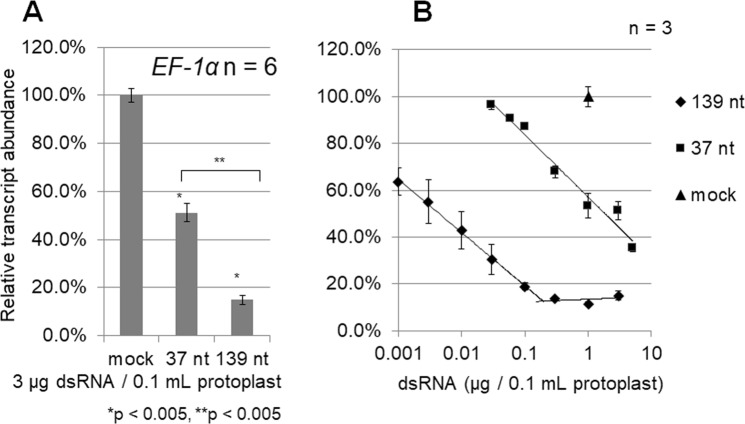
A long dsRNA of 139 nt is much more effective (approximately 400-fold) at inducing RNAi of the endogenous *EF-1α* gene than a short dsRNA of 37 nt. (**A**) Direct transfer of dsRNAs successfully induced RNAi of the endogenous *EF-1α* gene, which is a typical house-keeping gene^31^. Protoplasts in 100 µL (10^6^ to 10^7^ cells/mL) were transfected without (mock) or with 3 µg of short (37 nt) or long (139 nt) dsRNA against the *EF-1α* gene (RNAi). Protoplasts were collected 2 days after transfection. The abundance of *EF-1α* transcripts in dsRNA-transfected protoplasts relative to that in mock-transfected protoplasts (100%) was determined by qRT-PCR and normalized to the abundance of *ACT2* transcripts. Error bars indicate the standard errors of six biological replicates, and p-values were determined using the Student’s *t*-test. Single asterisks (*) indicate significant differences between mock- and dsRNA-transfected (37 nt and 139 nt) protoplasts, and double asterisk (**) indicates a significant difference between 37 nt and 139 nt dsRNA-transfected protoplasts. (**B**) Dose dependence of dsRNAs for inducing RNAi of the endogenous *EF-1α* gene in protoplasts was examined by transfecting protoplasts with varying amounts (0.001 to 5 µg) of 37-nt or 139-nt dsRNAs corresponding to the *EF-1α* gene. The relative abundance of *EF-1α* transcripts in protoplasts was determined by qRT-PCR and normalized to the abundance of *ACT2* transcripts. The amount of 139-nt dsRNA needed to reduce the level of *EF-1α* transcripts (RNAi) by 50% was estimated approximately 0.0045 µg, whereas the amount of 37-nt dsRNA needed to reduce the level of *EF-1α* transcripts (RNAi) by 50% was estimated at 1.84 µg. Error bars indicate the standard error of three biological replicates.

**Figure 5:**
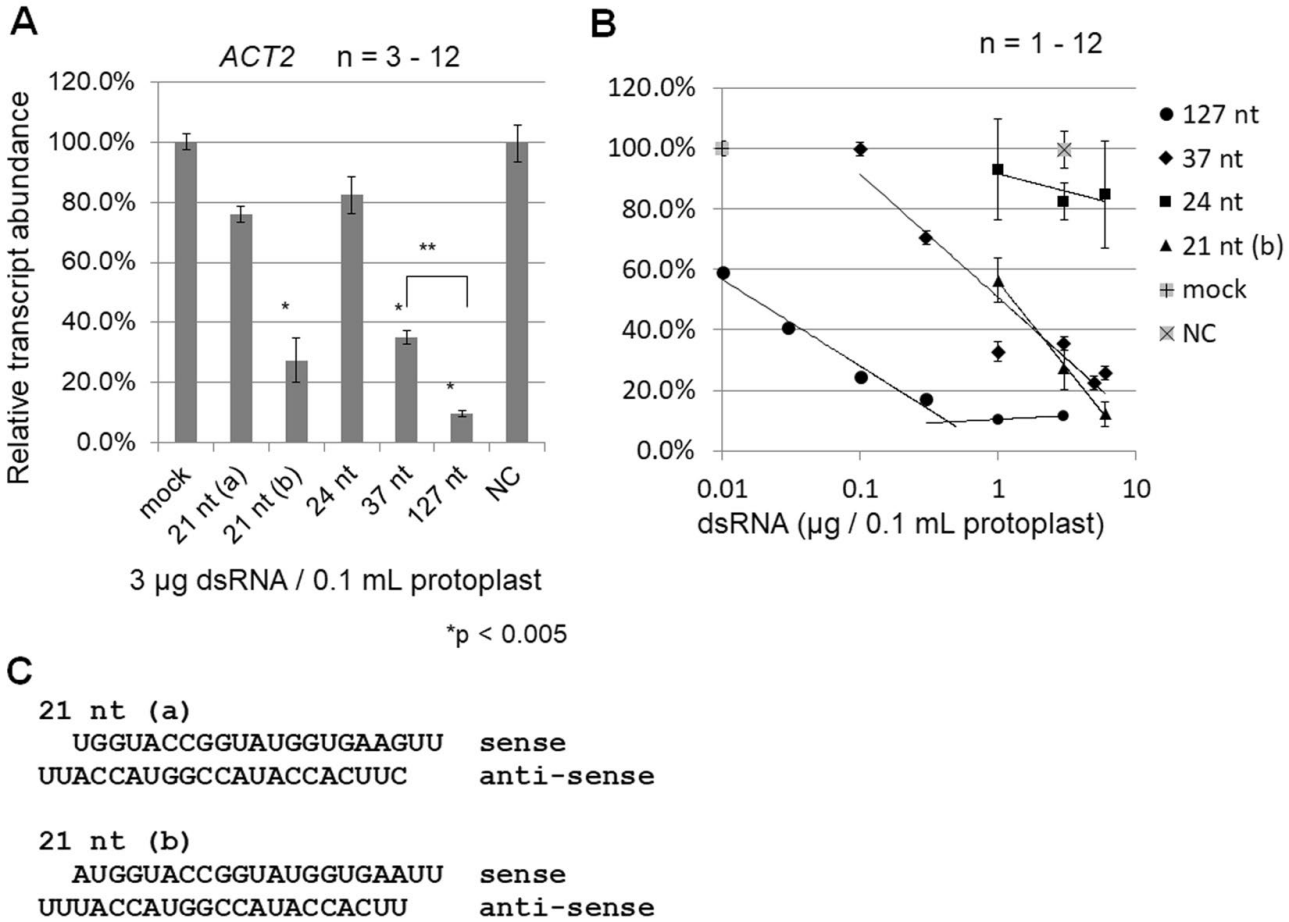
A long dsRNA of 127 nt is much more effective at inducing RNAi of the endogenous *ACT2* gene than 21-nt dsRNAs (siRNA duplexes). (**A**) Protoplasts in 100 µL (10^6^ to 10^7^ cells/mL) were transfected without (mock) or with 3 µg of various-sized dsRNAs corresponding to the *ACT2* gene (RNAi) or 130-nt dsRNA corresponding to the GFP gene (NC, negative control). Protoplasts were collected 2 days after transfection. The abundance of *ACT2* transcripts in dsRNA-transfected protoplasts relative to that in mock-transfected protoplasts (100%) was determined by qRT-PCR and normalized to the level of *EF-1α* transcripts. Error bars indicate the standard error of 3 to 12 biological replicates, and p-values were determined using the Student’s *t*-test. Single asterisks (*) indicate significant differences between mock- and dsRNA-transfected protoplasts (21 nt [b], 37 nt and 127 nt), and double asterisk (**) indicates a significant difference between 37-nt and 127-nt dsRNA-transfected protoplasts. (**B**) Dose dependence of dsRNAs for inducing RNAi of the *ACT2* gene. Protoplasts were transfected with varying amounts (0.01 to 5 µg) of dsRNAs of 21 to 127 nt in length corresponding to the endogenous *ACT2* gene. Nucleotide sequences of two 21-nt dsRNAs with different nucleotide pairs at each end (21 nt [a] and [b]) are shown in (**C**).

As we demonstrated that DCL3 preferentially cleaves short dsRNAs of <50 nt into 24-nt dsRNAs and DCL4 preferentially cleaves long dsRNAs of >50 nt into 21-nt dsRNAs *in vitro*^21^, we examined the efficacies of various-sized dsRNAs for inducing RNAi by directly transferring dsRNAs into protoplasts. We synthesized 37-nt and approximately 130-nt dsRNAs from Green Fluorescent Protein (GFP), *Arabidopsis* ACTIN2 (*ACT2*), and ELONGATION FACTOR-1α (*EF-1α*) complementary DNAs (cDNAs) *in vitro* and used them to transfect protoplasts prepared from wild-type (WT) and GFP-over-expressing *Arabidopsis* seedlings (Figs 3–5). Two days after transfection, the efficacies of the dsRNAs for inducing RNAi were evaluated microscopically determining the fluorescence intensity of protoplasts (Fig. 3A) using ImageJ software (Fig. 3B). In addition, relative mRNA accumulation was assessed using quantitative real-time PCR (qRT-PCR) (Figs 3C–5).

In GFP-overexpressing protoplasts transfected with GFP dsRNAs, the green fluorescence of the protoplasts (Fig. 3A,B) and the relative abundance of GFP transcripts (Fig. 3C) were reduced, but only in protoplasts transfected with the 130-nt GFP dsRNA. In WT protoplasts transfected with *EF-1α* and *ACT2* dsRNAs (Figs 4A and 5A), long dsRNAs of approximately 130 nt induced RNAi much more effectively than short dsRNAs of 21 to 37 nt. In all three experiments in which different dsRNAs were transfected (Figs 3–5), the abundance of target transcripts in protoplasts transfected with long dsRNAs relative to the abundance in mock-transfected protoplasts was lowest (about 10 to 15%). Knockdown levels of target mRNAs (10 to 15%) in protoplasts transfected with 3 µg of long dsRNAs were high relative to previous reports involving this protoplast RNAi system^28,29^. However, there was no or little decrease in the relative abundance of target transcripts in protoplasts transfected with short dsRNAs of 21, 24 or 37 nt relative to protoplasts transfected with long dsRNAs.

Although these three genes (particularly *GFP*, which is driven by the cauliflower mosaic virus 35S promoter) are highly and constitutively expressed^30,31^, direct transfer of long dsRNAs into protoplasts efficiently reduced the expression of these genes to approximately 10% of the control (Figs 3–5).

### Dose dependence of transfected dsRNAs for inducing RNAi

As the transfection of protoplasts with 3 µg of long dsRNAs seemed sufficient to induce efficient knockdown of target gene expression (10 to 15%), the dose dependence of transfected dsRNAs for inducing RNAi was examined by directly transfecting WT protoplasts with varying amounts of *EF-1α* (Fig. 4B) and *ACT2* (Fig. 5B) dsRNAs.

In both transfection experiments (Figs 4B and 5B), approximately 300 ng of 130-nt dsRNAs were sufficient to reduce transcript levels to <20%, and transfections of longer dsRNAs appeared to saturate the knock-down of gene expression. From the results shown in Fig. 4B, approximately 4.5 ng of 139-nt *EF-1α* dsRNA was estimated to be sufficient to induce a 50% reduction in *EF-1α* transcript level, but approximately 1.8 µg of 37-nt *EF-1α* dsRNA would be needed to reduce the transcript level by 50%. The efficacy of the 139-nt dsRNA for inducing RNAi was thus 400-fold greater than that of the 37-nt dsRNA (Fig. 4B). The same calculation was carried out using the data shown in Fig. 5B (*ACT2*) and revealed that the efficacy of the 127-nt *ACT2* dsRNA for inducing RNAi was 60-fold greater than that of the 37-nt *ACT2* dsRNA. Therefore, regardless of whether target genes are exogenous (GFP) or endogenous (*EF-1α* and *ACT2*), the direct transfer of long dsRNAs of approximately 130 nt efficiently induces RNAi in protoplasts.

These results are consistent with our previous results demonstrating that DCL4 cleaves long dsRNAs >50 nt efficiently and 37-nt dsRNAs slightly *in vitro*^21^. They are also consistent with the results shown in Fig. 2 that cleavage products of 21-nt by DCL4 were detected from 130-nt dsRNAs but not from 37-nt dsRNAs in protoplasts. As DCL4 is essential for RNAi in plants^32,33^ and long dsRNAs are preferred substrates for DCL4 in plant cells (protoplasts), they are able to induce RNAi more efficiently than short dsRNAs. Because short dsRNAs of 37 nt did induce RNAi ineffectively (Figs 4 and 5), 21-nt siRNAs must be produced from 37-nt dsRNAs by DCL4 in protoplasts but they were not detected (Fig. 2).

### Cleavage of long dsRNAs by DCL4 is essential for efficient induction of RNAi in protoplasts

Short dsRNAs (siRNA duplexes) of 21 to 24 nt are produced from long dsRNAs by three Dicers (DCL2, DCL3 and DCL4) in plants, so the direct transfer of 21-nt dsRNAs into protoplasts must induce RNAi without Dicer activity. As 21-nt siRNAs produced by DCL4 generally induce RNAi (mRNA cleavage) but 24-nt siRNAs produced by DCL3 induce RNA-directed DNA methylation (RdDM), we transfected protoplasts with 21-nt dsRNAs (RNAi) and 24-nt dsRNAs (RdDM).

The 21-nt dsRNA (b) induced RNAi more efficiently than 21-nt dsRNA (a) and 24-nt dsRNA (Fig. 5). It is therefore reasonable to conclude that the 21-nt dsRNA (b) is more efficient than the 24-nt dsRNA, because 21-nt siRNAs induce RNAi and 24-nt siRNAs induce RdDM. Interestingly, the efficacy of two different 21-nt dsRNAs for inducing RNAi differed depending on their sequences at both ends of each dsRNA (compare 21 nt [a] with 21 nt [b] in Fig. 5C). If the guide-strand selection rule for AGO proteins (demonstrated primarily in a biochemical study using fruit fly extracts^34^) is applicable to plant AGO proteins^35^, the 21-nt dsRNA (a) sense strand could be preferentially loaded onto AGO1 as the guide strand (Fig. 5C). However, AGO1 with the 21-nt dsRNA (a) sense strand cannot slice *ACT2* mRNA. The 21-nt dsRNA (b) antisense strand can be loaded onto AGO1, and this complex can slice *ACT2* mRNA. Furthermore, as the nucleotide at the 5′-ends of the antisense strands of 21-nt dsRNA (a) and (b) are cytidine and uridine, respectively, they can be loaded onto AGO5 and AGO1, respectively^36,37^. Experiments examining this interesting relationship between 21-nt dsRNA nucleotide sequences and their efficacy for inducing RNAi are now in progress. Even though the 21 nt dsRNA (b) was more efficient than the 21-nt dsRNA (a), the long dsRNA of approximately 130 nt induced RNAi much more efficiently than the 21-nt dsRNA (b) (Fig. 5B).

The 127-nt *ACT2* dsRNA induced RNAi about 100-fold more efficiently than the 21-nt (b) dsRNA (Fig. 5B), suggesting that cleavage of long dsRNAs into 21-nt dsRNAs (siRNA duplexes) by DCL4 is essential for efficient induction of RNAi in plants. RNAi enhancement via the cleavage of dsRNA substrates by Dicer enzymes has been reported in mammalian cells^13^, in which it was demonstrated that dsRNAs of 25 to 30 nt induce RNAi up to 100-fold more potently than 21-nt dsRNAs in human cells. In plant protoplasts (Fig. 5A,B), however, 37-nt dsRNAs were as efficient as the 21-nt dsRNA (21 nt [b]). The reason for the ineffective induction of RNAi in plant cells by 37-nt dsRNAs (Figs 3–5) may be the ineffective cleavage of 37-nt dsRNAs by DCL4 in protoplasts (Fig. 2) and *in vitro*^21^.

### dsRNA-induced RNAi in protoplasts depends primarily on DCL4

**Figure 6:**
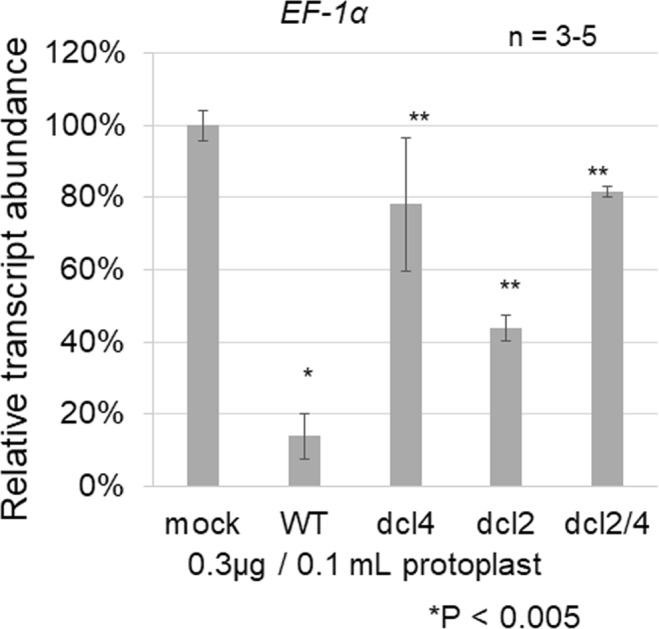
Efficiency of RNAi in *dcl* mutants. A 0.1-mL volume of protoplasts (10^6^ to 10^7^ cells/mL) prepared from *Arabidopsis* WT or *dcl* mutant seedlings were transfected with 0.3 µg of 139-nt *EF-1α* dsRNA. Protoplasts were collected 2 days after transfection, and then the abundance of *EF-1α* transcripts in transfected protoplasts relative to that in mock-transfected protoplasts (100%) was determined by qRT-PCR and normalized to the level of *ACT2* transcripts. Error bars indicate the standard error of three to five biological replicates, and p-values were determined using the Student’s *t*-test. Single asterisk (*) indicates significant difference between mock- and 139-nt dsRNA-transfected WT protoplasts, and double asterisks (**) indicate significant differences between WT and the three mutant protoplasts.

To confirm the involvement of DCL4 in inducing RNAi in protoplasts, we transfected protoplasts prepared from *dcl*-mutant seedlings. In experiments in which 0.3 µg of 139-nt *EF-1α* dsRNA was transfected into protoplasts prepared from seedlings of WT, *dcl2* single, *dcl4* single, and *dcl2dcl4* double mutants, less RNAi was induced in the *dcl4* and *dcl2dcl4* protoplasts, with the *EF-1α* transcript abundance reduced to only approximately 80% of the control (Fig. 6A), indicating that DCL4 is primarily involved in inducing RNAi in protoplasts, as shown in previous genetic studies^32,33^. The efficiency of RNAi induction in *dcl2* protoplasts (approximately 40%) was intermediate between WT (approximately 10%) and *dcl4* (approximately 80%) (Fig. 6). The results shown in Fig. 6 and those of previous genetic studies by another group^26,38,39^ suggest that DCL2 plays a role in inducing RNAi in the absence of DCL4.

## Discussion

The relationship between dsRNA length and efficacy in inducing RNAi in plants has received little research attention to date because the direct transfer of dsRNAs into intact plant cells is very difficult due to the cell wall. Here, we demonstrated for the first time that long DCL4-substrate dsRNAs >130 nt induce RNAi up to 400-fold more potently than short dsRNAs of 21 or 37 nt and that the cleavage of long dsRNAs by DCL4 is essential for efficient induction of RNAi in plant cells (Supplementary Fig. S1). DCL4 preferentially cleaves long dsRNAs of approximately 130 nt into 21-nt siRNAs (Fig. 2) that efficiently induce RNAi in *Arabidopsis* protoplasts (Figs 3–5). This is consistent with the results of our previous *in vitro* biochemical analysis^21^.

The current design for comparing the efficacies of various-sized dsRNAs for inducing RNAi might not have been perfect, because various 21-nt siRNAs with different sequences could be produced from long dsRNAs by DCL4 but uniform 21-nt siRNAs with a specific sequence could be produced from 37-nt dsRNAs. That is, the efficacies of various dsRNAs for inducing RNAi may be dependent on the size of dsRNAs but also the nucleotide sequences of dsRNAs. However, the efficient induction of RNAi by long DCL4-substrate dsRNAs observed in one exogenous gene (*GFP*) and two endogenous genes (*ACT2* and *EF-1α*) (Figs 3–5) suggests that this is a common mechanism (phenomenon) in plant cells, regardless of the origin and nucleotide sequence of target gene.

As we could not measure the transfection efficiencies of dsRNAs in protoplasts in the present study, transfection efficiency was ignored with respect to the efficacy of dsRNAs for inducing RNAi. This is because transfecting long dsRNAs into protoplasts may be less efficient than transfection of short dsRNAs, and the efficacy of long dsRNAs for inducing RNAi may be underestimated.

The dsRNA-cleaving activity of DCL3, which cleaves 37-nt dsRNA into 24-nt RNA, can be readily detected in crude extracts prepared from 2-week-old *Arabidopsis* seedlings using an *in vitro* biochemical assay^21,22^. However, no products of cleavage of 37-nt dsRNAs by DCL3 were detected in protoplasts in the present study, although products of cleavage of 130-nt dsRNAs by DCL4 were detected (Fig. 2). The subcellular localizations of DCL3 and DCL4 may explain this discrepancy, as DCL3 is localized and functions in the nucleus^40,41^, whereas DCL4 is localized primarily in the cytoplasm^42,43^. Transfection of inducer dsRNAs into the cytoplasm in the protoplast, as occurs in the RNAi system we used, could render it impossible to detect DCL3 activity under the conditions used in our experiments.

DCL2 and DCL4 cleave viral and transgene-derived dsRNAs into 22- and 21-nt siRNAs, respectively, and they function redundantly and hierarchically in virus resistance and RNAi^30,31,36,37^. DCL4 functions as a primary Dicer (DCL) for virus resistance and RNAi, whereas DCL2 serves as a functional backup in the absence of DCL4. The results shown in Fig. 6 indicate that DCL2 and DCL4 also function redundantly and hierarchically in RNAi in plant protoplasts.

Long dsRNAs of 130 nt exhibited greater efficiency for inducing RNAi than 21-nt dsRNAs, which are not necessary for DCL4 activity to induce RNAi. Although a similar phenomenon has been reported in mammalian cells^13^, this is the first such report in plants. Kim *et al*. noted that the enhanced induction of RNAi by dsiRNAs in mammalian cells results from the close link between Dicer cleavage of dsRNAs into siRNAs and loading of cleaved siRNAs onto AGO proteins^13^. Our results suggest that a similar link between Dicer cleavage and AGO loading exists in plant cells and that DCL4-cleaved siRNAs (i.e., not free siRNAs) may also efficiently load onto AGOs to induce RNAi in plant cells.

Short dsiRNAs of 25 to 30 nt are typically used for RNAi experiments in mammalian cells because they do not activate the innate immune response, including the interferon pathway^9,10,13^. In contrast, in this study, we demonstrated that short dsRNAs of 37 nt are not potent inducer of RNAi in plant cells because they are not cleaved by DCL4, whereas long dsRNAs of 130 nt potently induce RNAi because they are preferentially cleaved by DCL4. Therefore, the results of RNAi experiments in plant protoplasts reported here are consistent with our previous results regarding the substrate specificities of DCL3 and DCL4 *in vitro*^21^. Moreover, these results suggest that DCL3 and DCL4 function according to their substrate specificities *in vivo*. In mammals, long dsRNAs >30 nt activate the innate immune system^9,10^, whereas short Dicer-substrate dsRNAs of 25 to 30 nt efficiently induce the RNAi pathway without activating an immune response^12,13^. Although long DCL4-substrate dsRNAs efficiently induce RNAi in plants, short DCL3-substrate dsRNAs of 30 to 37 nt might induce RdDM but not RNAi^21,24,25^. Experiments examining the induction of RdDM by the direct transfer of short dsRNAs into protoplasts are now in progress.

## Methods

### Plant material and growth conditions

WT and *dcl* mutant *A*. *thaliana* (Columbia ecotype) seeds were sterilized in 70% ethanol, and 6% sodium hypochlorite with 0.1% Tween 20. Seeds were sown on Murashige and Skoog (MS) medium supplemented with 1% sucrose and 0.7% agar and then kept for 48 h at 4 °C in the dark for stratification. Seeds were germinated and seedlings were grown in a 16-h-light photoperiod at 22 °C.

Seeds of *dcl2-1* (CS16389) and *dcl3-1* (SALK_005512) were kindly provided by the Arabidopsis Biological Resource Center (ABRC, USA), and seeds of *dcl4-2* (GABI 160G05) were kindly provided by the Genomanalyse im Biologischen System Pflanze (Germany). Seeds of a transgenic *Arabidopsis* variety that over expresses *GFP* were kindly provided by Dr. Yasuo Niwa (University of Shizuoka, Japan)^30^.

### Isolation of protoplasts from *Arabidopsis* seedlings

Protoplasts were isolated from *Arabidopsis* seedlings using a modified method of Zhai *et al*.^28^. Approximately 2 g of 2-week-old *Arabidopsis* seedlings were sliced with a razor blade into stripes of approximately 1 mm in 15 mL of filter-sterilized TVL solution (0.3 M sorbitol and 50 mM CaCl_2_). Next, 20 mL of enzyme solution (0.5 M sucrose, 20 mM MES-KOH (pH 5.7), 20 mM CaCl_2_, 40 mM KCl, 1% cellulase [Onozuka R-10], and 1% Macerozyme [R10]) was added to the sliced seedlings, and the mixture was agitated at 35 rpm in the dark at 22 °C. After 16 to 18 h, the released protoplasts were sieved through a nylon mesh (80 µm) and transferred into 50-mL centrifuge tubes. Protoplasts and plant debris retained on the nylon mesh were gently sieved-through one more time by washing the mesh with 5 mL of W5 solution (0.1% glucose, 0.08% KCl, 0.9% NaCl, 1.84% CaCl_2_, and 2 mM MES-KOH [pH 5.7]). Sieved-through protoplasts were combined in a 50-mL centrifuge tube, overlaid with 10 mL of W5 solution, and centrifuged for 20 min at 100 g at 22 °C. Approximately 10 mL of the protoplast fraction was collected from the interface of the enzyme and W5 solutions (Fig. 1). Protoplasts were washed twice with 15 mL of W5 solution and suspended in 1 to 2 mL of W5 solution. The concentration of protoplasts was measured by cell counting using a hemocytometer.

### Preparation of dsRNAs

DNA templates for short dsRNAs <37 nt were designed from cDNA sequences of *Arabidopsis* ACT2 (At3g18780), *Arabidopsis* EF-1α (At1g07920), and GFP, engineered to contain the minimal T7-RNA polymerase promoter sequence (TAATACGACTCACTATAGGG) at both the 5′ and 3′ ends, and then synthesized by Sigma-Aldrich (Japan). DNA templates for long dsRNAs were synthesized by PCR using KOD-plus DNA polymerase (Toyobo, Japan) from plasmids containing an *Arabidopsis* cDNA. Nucleotide sequences of DNA templates for short dsRNA syntheses and primers used to amplify DNAs of target genes for long dsRNA syntheses are listed in Table S1. dsRNAs were synthesized using the *in vitro* T7 Transcription kit for siRNA synthesis (Takara, Japan) according to the manufacturer’s recommendations (http://catalog.takarabio.co.jp/product/basic_info.php?unitid=U100004288). A 2-nt 3′ overhang of dsRNAs was made using RNase T1, and DNA templates were removed with RNase-free DNase I (Takara). dsRNAs were extracted by phenol/chloroform/isoamyl alcohol (25:24:1), precipitated with an equal volume of 5 M ammonium acetate and 4 volumes of ethanol, and reconstituted with ST buffer (10 mM Tris-HCl [pH 7.5] and 100 mM NaCl). The quality and quantity of synthesized dsRNAs were determined by agarose gel electrophoresis (see Supplementary Fig. S2) and UV spectrophotometry (NanoDrop Lite Microlitre spectrophotometer, Labtech International Ltd., UK).

### Transfection of protoplasts with dsRNAs

Protoplasts were transfected using a modified method of Jung *et al*.^29^. Briefly, protoplasts in W5 solution were incubated on ice for 30 min, after which the W5 solution was discarded, and protoplasts were resuspended in MMG solution (4 mM MES-KOH [pH5.7], 0.4 M mannitol, and 15 mM MgCl_2_) to a final concentration of 10^6^ to 10^7^ protoplasts/mL. Aliquots of protoplasts (100 µL) were transferred into a 2-mL round-bottom microcentrifuge tube and mixed gently with dsRNA (0.001 to 5 µg) and 110 µL of PEG-calcium solution (40% PEG-4000, 0.2 M mannitol, and 100 mM CaCl_2_). In negative control transfections, dsRNA was omitted, and an equivalent volume of deionized, sterile water was used for mock-transfection. Protoplasts were mixed with PEG-calcium solution by gently tapping the tube and incubated for 7 min at room temperature. Transfection was terminated by diluting the mixture with 600 µL of W5 solution. Transfected protoplasts were collected by centrifugation for 2 min at 100 *g*, resuspended in 1 mL of W5 solution, and kept in the dark for 2 days.

### qRT-PCR

Transfected protoplasts (10^6^ to 10^7^ cells/mL) were collected by centrifugation for 2 to 5 min at 100 *g*. Total RNAs were extracted with 1 mL of TRIzol reagent following the manufacturer’s protocol (Thermo Fisher Scientific, Japan). cDNAs were produced from total RNAs using a PrimeScript RT reagent kit with gDNA Eraser (Takara), and qRT-PCR was performed using a Thermal Cycler Dice Real Time System with a SYBR *Premix Ex Taq* II kit (Takara). Primers for qRT-PCR are listed in Table S2. The effectiveness of RNAi was evaluated by comparing the relative abundance of RNAi-target transcripts to transcripts of *Arabidopsis* house-keeping genes, such as *ACT2* and *EF-1α*^31^.

### Monitoring of Dicer (dsRNA-cleaving) activity in protoplasts

Methods for the preparation of 5′-^32^P-labelled short dsRNAs of 37 nt and [α-^32^P]-UTP-labeled long dsRNAs of approximately 130 nt and 500 nt were described previously (Table S3)^20,21^. Nucleotide sequences of 37-nt dsRNAs are shown in Table S3. Protoplasts were transfected with ^32^P-labelled short and long dsRNAs, and then incubated for 2 h at room temperature. Total RNAs were extracted from protoplasts using phenol/chloroform/isoamyl alcohol (25:24:1), precipitated with ethanol, separated using 15% denaturing PAGE with 8 M urea, and detected by autoradiography (Typhoon FLA 7000 image analyser, GE Healthcare).

### Fluorescence intensity quantification

GFP-expressing protoplasts were observed under light microscopy (IX71, Olympus, Tokyo, Japan) with a fluorescence excitation filter (470–495 nm) and emission filter (510–550 nm). Fluorescence intensities of more than 20 protoplasts were quantified as gray values, and the area of each protoplast was calculated using ImageJ software (https://imagej.nih.gov/ij/). Relative fluorescence intensities were calculated as gray values normalized to the average protoplast area; three photographs containing more than 20 protoplasts were analysed.

## Supplementary information

Dataset 1
