# Supplementary material for: Long DCL4-substrate dsRNAs efficiently induce RNA interference in plant cells
Source: Sci Rep. 2019 May 6;9:6920. doi: 10.1038/s41598-019-43443-9 (PMC6502814; doi:10.1038/s41598-019-43443-9)
Supplement: Supplementary file 1 — Dataset 1 [file 41598_2019_43443_MOESM1_ESM.pdf]

Supplementary Materials

Long DCL4-substrate dsRNAs efficiently induce RNA interference in plant cells

Sayaka Kakiyama[1], Midori Tabara[1], Yuki Nishibori[1], Hiromitsu Moriyama[1] and Toshiyuki Fukuhara[1, 2] *

[1]Department of Applied Biological Sciences and [2]Institute of Global Innovation Research, Tokyo University of Agriculture and Technology, 3-5-8 Saiwaicho, Fuchu, Tokyo 183-8509, Japan

*Corresponding author: fuku@cc.tuat.ac.jp

Supplementary Tables

Table S1. Primers for dsRNA preparation

Primer	dsRNA	sequence	dsRNA size
T7GFP1	GFP 100 nt	TCACTAATACGACTCACTATAGGGAGCTGACCCTGAAGTTCATCT	130
T7GFP2		TCACTAATACGACTCACTATAGGGAAGTCGTGCTGCTTCATGT	
GFP1		AAAGCTGACCCTGAAGTTCATCT	
GFP2		AAGAAGTCGTGCTGCTTCATGT	
T7GFP3	GFP 500 nt	TCACTAATACGACTCACTATAGGGACGTAAACGGCCACAAGTTC	501
T7GFP4		TCACTAATACGACTCACTATAGGGGTGTTCTGCTGGTAGTG	
GFP3		AAACGTAAACGGCCACAAGTTC	
GFP4		AAGGGGTGTTCTGCTGGTAGTG	
GFP37-1	GFP 37 nt U	TCACTAATACGACTCACTATAGGGTCTGCACCACCGGCAAGCTGCCCGTGCCCTGGCCCTT	37
GFP37-2		AAGGGCCAGGGCACGGGCAGCTTGCCGGTGGTGCAGACCCTATAGTGAGTCGTATTAGTGA	
GFP37-3		TCACTAATACGACTCACTATAGGGCCAGGGCACGGGCAGCTTGCCGGTGGTGCAGATT	
GFP37-4		AATCTGCACCACCGGCAAGCTGCCCGTGCCCTGGCCCTATAGTGAGTCGTATTAGTGA	
GFP37-5	GFP 37 nt A	TCACTAATACGACTCACTATAGGGATCTGCACCACCGGCAAGCTGCCCGTGCCCTGGCCTT	37
GFP37-6		AAGGCCAGGGCACGGGCAGCTTGCCGGTGGTGCAGATCCCTATAGTGAGTCGTATTAGTGA	
GFP37-7		TCACTAATACGACTCACTATAGGGCCAGGGCACGGGCAGCTTGCCGGTGGTGCAGATTT	
GFP37-8		AAATCTGCACCACCGGCAAGCTGCCCGTGCCCTGGCCCTATAGTGAGTCGTATTAGTGA	
GFP24-1	GFP 24 nt A	TCACTAATACGACTCACTATAGGGATCTGCACCACCGGCAAGCTGCTT	24
GFP24-2		AAGCAGCTTGCCGGTGGTGCAGATCCCTATAGTGAGTCGTATTAGTGA	
GFP24-3		TCACTAATACGACTCACTATAGGGCAGCTTGCCGGTGGTGCAGATTT	
GFP24-4		AAATCTGCACCACCGGCAAGCTGCCCTATAGTGAGTCGTATTAGTGA	

T7GFP21-1	GFP 21 nt	TCACTAATACGACTCACTATAGGGTCTGCACCACCGGCAAGCTTT	21
T7GFP21-2		AAAGCTTGCCGGTGGTGCAGACCCTATAGTGAGTCGTATTAGTGA	
T7GFP21-3		TCACTAATACGACTCACTATAGGGAGCTTGCCGGTGGTGCAGATT	
T7GFP21-4		AATCTGCACCACCGGCAAGCTCCCTATAGTGAGTCGTATTAGTGA	

T7ACT2-100-1	ACT2 100 nt	TCACTAATACGACTCACTATAGGGAAAAATGGCTGAGGCTGATG	127
T7ACT2-100-2		TCACTAATACGACTCACTATAGGGTTGGCCTACCAACAACACTG	
ACT2-100-1		AAAAAAATGGCTGAGGCTGATG	
ACT2-100-2		AATTGGCCTACCAACAACACTG	
ACT2-37-1	ACT2 37 nt	TCACTAATACGACTCACTATAGGGACAATGGTACCGGTATGGTGAAGGCTGGATTTGCATT	37
ACT2-37-2		AATGCAAATCCAGCCTTCACCATACCGGTACCATTGTCCCTATAGTGAGTCGTATTAGTGA	
ACT2-37-3		TCACTAATACGACTCACTATAGGGTGCAAATCCAGCCTTCACCATACCGGTACCATTGTTT	
ACT2-37-4		AAACAATGGTACCGGTATGGTGAAGGCTGGATTTGCACCCTATAGTGAGTCGTATTAGTGA	
T7ACT2-24-1	ACT2 24 nt	TCACTAATACGACTCACTATAGGGACAATGGTACCGGTATGGTGAATT	24
T7ACT2-24-2		AATTCACCATACCGGTACCATTGTCCCTATAGTGAGTCGTATTAGTGA	
T7ACT2-24-3		TCACTAATACGACTCACTATAGGGTTCACCATACCGGTACCATTGTTT	
T7ACT2-24-4		AAACAATGGTACCGGTATGGTGAACCCTATAGTGAGTCGTATTAGTGA	
T7ACT2-21-1	ACT2 21 nt a	TCACTAATACGACTCACTATAGGGTGGTACCGGTATGGTGAAGTT	21
T7ACT2-21-2		AACTTCACCATACCGGTACCACCCTATAGTGAGTCGTATTAGTGA	
T7ACT2-21-3		TCACTAATACGACTCACTATAGGGCTTCACCATACCGGTACCATT	
T7ACT2-21-4		AATGGTACCGGTATGGTGAAGCCCTATAGTGAGTCGTATTAGTGA	
T7ACT2-21.2-1	ACT2 21 nt b	TCACTAATACGACTCACTATAGGGATGGTACCGGTATGGTGAATT	21
T7ACT2-21.2-2		AATTCACCATACCGGTACCATCCCTATAGTGAGTCGTATTAGTGA	
T7ACT2-21.2-3		TCACTAATACGACTCACTATAGGGTTCACCATACCGGTACCATTT	
T7ACT2-21.2-4		AAATGGTACCGGTATGGTGAACCCTATAGTGAGTCGTATTAGTGA	

T7EF1A-100-1	EF1A 100 nt	TCACTAATACGACTCACTATAGGGTACCCACCACTTGGACGTTT	139
T7EF1A-100-2		TCACTAATACGACTCACTATAGGGTTCACTTCGCACCCTTCTT	
EF1A-100-1		AATACCCACCACTTGGACGTTT	
EF1A-100-2		AAGTTCACTTCGCACCCTTCTT	
EF1A-37-1	EF1A 37 nt	TCACTAATACGACTCACTATAGGGACAAGAAGGACCCAACCGGAGCC AAGGTTACCAAGTT	37
EF1A-37-2		AACTTGGTAACCTTGGCTCCGGTTGGGTCCTTCTTGTCCCTATAGTGA GTCGTATTAGTGA	
EF1A-37-3		TCACTAATACGACTCACTATAGGGCTTGGTAACCTTGGCTCCGGTTGG GTCCTTCTTGTTT	
EF1A-37-4		AAACAAGAAGGACCCAACCGGAGCCAAGGTTACCAAGCCCTATAGTG AGTCGTATTAGTGA	

Table S2. Primers for qRT-PCR

primer	target gene	sequence
GFP1-1F	GFP	GCAAGCTGACCCTGAAGTT
GFP1-1R		GGTCTTGTAGTTGCCGTCGT
ACT2F	ACT2	TCCGCTCTTTCTTTCCAAGCTCA
ACT2-1R-2R		TCCTTCTGGTTCATCCCAAC
EF-1A F	EF1A	TGCCGCAGGTGAATCAAAGG
EF-1A R		CCCAATTACGAGAACAACGCTCTG

Table S3. RNA oligonucleotides for Dicer assay

RNA oligo	Target gene	Sequence
GFP37As	GFP 37 nt	5′ AUCUGCACCACCGGCAAGCUGCCCGUGCCCUGGCCCA 3′
GFP37Aas		3′ AGUAGACGUGGUGGCCGUUCGACGGGCACGGGACCGG 5′
Actin37s	ACT2 37 nt	5′ AGAUCCUAACCGAGCGUGGUUACUCUUUCACCACCAC 3′
Actin37as		3′CUUCUAGGAUUGGCUCGCACCAAUGAGAAAGUGGUGG 5'

Supplementary Figures

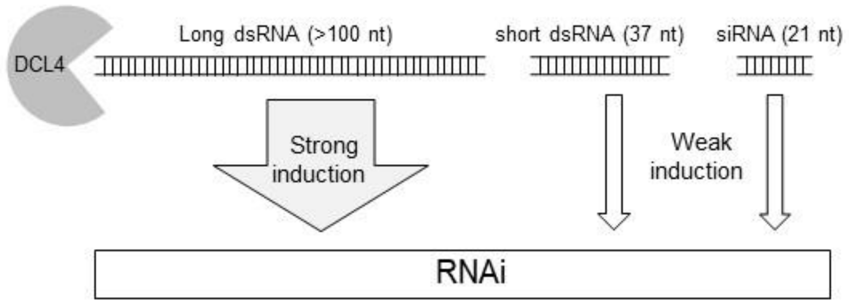

Figure S1. Graphical abstract.

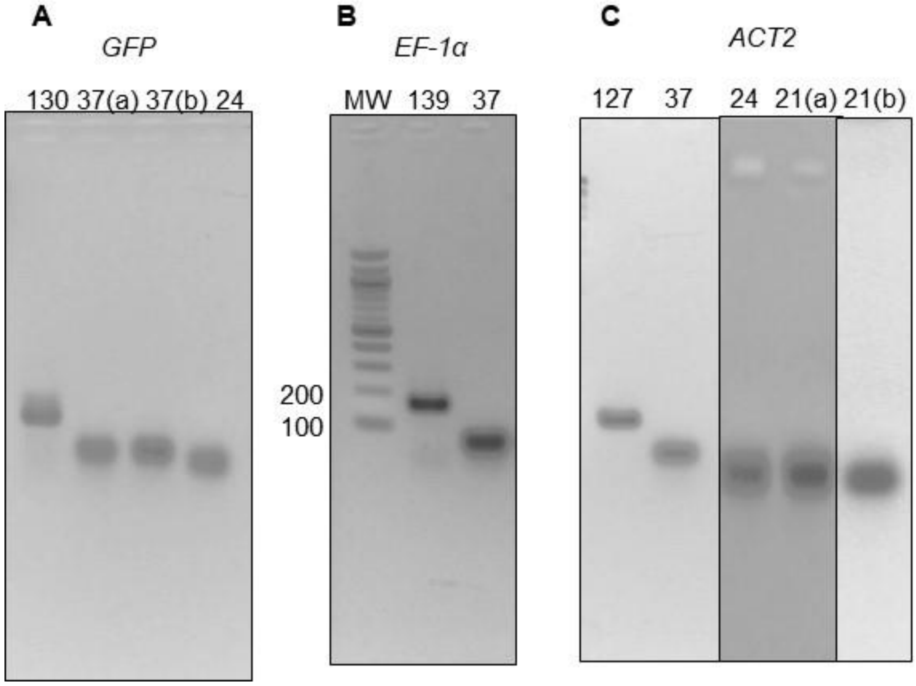

Figure S2. The quality and quantity of synthesized dsRNAs were determined by agarose gel electrophoresis. dsRNAs were stained by ethidium bromide. The quantity of dsRNAs was also measured by UV spectrophotometry.

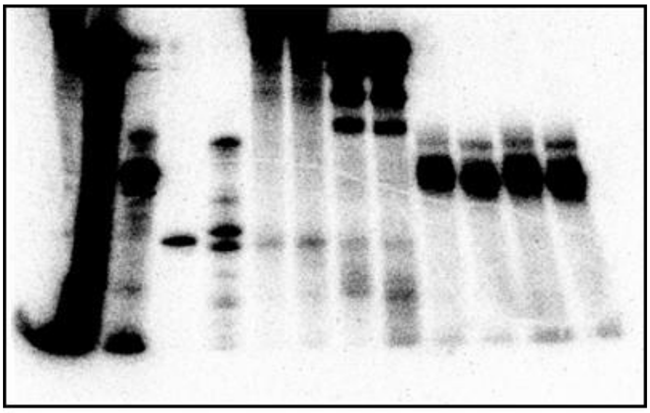

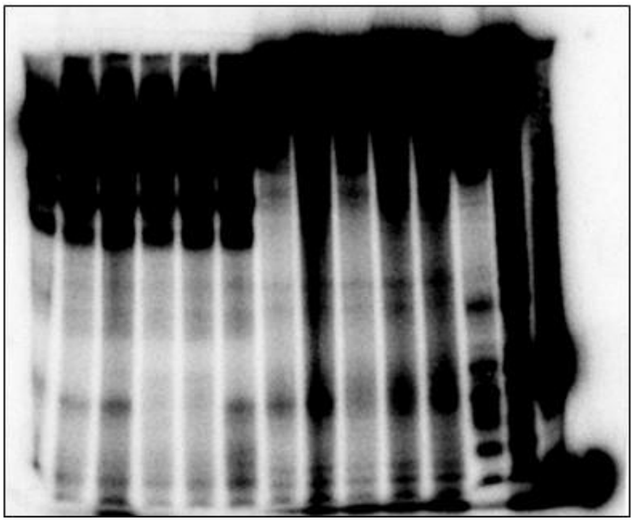

Figure S3. Original photographs, from which Figure 2 was made.
